# Eco-evolutionary significance of domesticated retroelements in microbial genomes

**DOI:** 10.1186/s13100-022-00262-6

**Published:** 2022-02-23

**Authors:** Blair G. Paul, A. Murat Eren

**Affiliations:** 1 Marine Biological Laboratory, Josephine Bay Paul Center, Woods Hole, MA USA; 2 Department of Medicine, University of Chicago, Chicago, IL USA

## Abstract

Since the first discovery of reverse transcriptase in bacteria, and later in archaea, bacterial and archaeal retroelements have been defined by their common enzyme that coordinates diverse functions. Yet, evolutionary refinement has produced distinct retroelements across the tree of microbial life that are perhaps best described in terms of their programmed RNA—a compact sequence that preserves core information for a sophisticated mechanism. From this perspective, reverse transcriptase has been selected as the modular tool for carrying out nature’s instructions in various RNA templates. Beneficial retroelements—those that can provide a fitness advantage to their host—evolved to their extant forms in a wide array of microorganisms and their viruses, spanning nearly all habitats. Within each specialized retroelement class, several universal features seem to be shared across diverse taxa, while specific functional and mechanistic insights are based on only a few model retroelement systems from clinical isolates. Currently, little is known about the diversity of cellular functions and ecological significance of retroelements across different biomes. With increasing availability of isolate, metagenome-assembled, and single-amplified genomes, the taxonomic and functional breadth of prokaryotic retroelements is coming into clearer view. This review explores the recently characterized classes of beneficial, yet accessory retroelements of bacteria and archaea. We describe how these specialized mechanisms exploit a form of fixed mobility, whereby the retroelements do not appear to proliferate selfishly throughout the genome. Moreover, we discuss computational approaches for systematic identification of retroelements from vast sequence repositories and highlight recent discoveries in terms of their apparent distribution and ecological significance in nature. Lastly, we present a new perspective on the eco-evolutionary significance of these genetic elements in marine bacteria and demonstrate approaches that enable the characterization of their environmental diversity through metagenomics.

**Supplementary Information:**

The online version contains supplementary material available at 10.1186/s13100-022-00262-6.

## Genetic memory in a template

Taking many different forms in microbial cells, RNA can transmit information from DNA to proteins, carry out biochemical reactions as ribozymes, or form ribonucleoprotein complexes that carry out various cellular functions. In retroelements, non-coding RNAs have an ability to preserve and transfer genetic information as a dynamic form of memory that is recalled in response to biological conflict. Several classes of genomic elements are found in bacteria and archaea that have the unique role of preserving and transmitting sequence information coupled to adversarial interactions among cells, or in response to environmental stress. To mitigate biological conflict, retroelements can store this memory in a genomic region that encodes a short RNA, which we refer to as a template. Whereas these templates themselves do not directly code for proteins, their sequences represent potential states of protein–protein or protein–ligand interaction. Beneficial retroelements are therefore defined by their essential RNA templates that preserve compact sequence information for a critical role in resolving conflict.

The capacity to store and transfer genetic memory through an RNA template is a common defining characteristic of two mechanisms that provide specific selective advantages to bacteria, archaea, and their viruses: diversity-generating retroelements (DGRs) and retrons. DGRs possess a template for sequence variation that is transmitted to a protein to enable interaction with a vast repertoire of ligands [1–3]. Retrons consist of a conserved template for a small DNA molecule, which interacts with immunity and effector proteins that were recently found to trigger growth arrest as a response to phage infection [4, 5].

Through direct sequencing of natural systems, we can view a snapshot of ongoing microbial evolution, including the role of specialized elements that likely underwent several dynamic stages of proliferation and refinement into their current genomic contexts. For any given retroelement, the timing of past mobility or potential for future proliferation is difficult to reconstruct. Retrons and DGRs are thought to have a complex evolutionary history of domestication from ancestral RTs towards functional coupling with proteins that offer independent cellular and viral properties. Here, we review these two classes of domesticated retroelements—retrons and diversity-generating retroelements—that have been refined to provide specific benefits to the host as a result of their ‘fixed’ mobility.

## Diversity-generating retroelements

Microbial genes adapt through evolutionary optimization that may involve combinations of multiple synergistic mutations. If many synergistic mutations exist for a gene, it may be impossible to reach a fitness optimum through stochastic mutation and selection alone; evolution to these optima might only be enabled by a specialized mechanism to rapidly explore simultaneous mutations. Hypermutation can spontaneously affect a microbial genome, where global phenomena include imperfect DNA damage repair, and errors in replication [6, 7]. While stochastic hypermutation is favored under environmental stress, it is generally reduced in well-adapted populations [8]. Alternatively, localized variation can result from targeted recombination, dynamic promoter inversion, or codon rewriting [9, 10]. Diversity-generating retroelements (DGRs) are drivers of hypermutation in target genes of bacteria, archaea, and temperate viruses [11, 12]. Through precise positioning of adenines in the template sequence, DGRs are able to target mutations at single-nucleotide resolution. The mutations will be overwritten whenever the DGR reactivates, suggesting that perhaps the phenotype of hypermutation is suppressed under stable cellular conditions to mitigate potential loss in fitness.

**Fig. 1:**
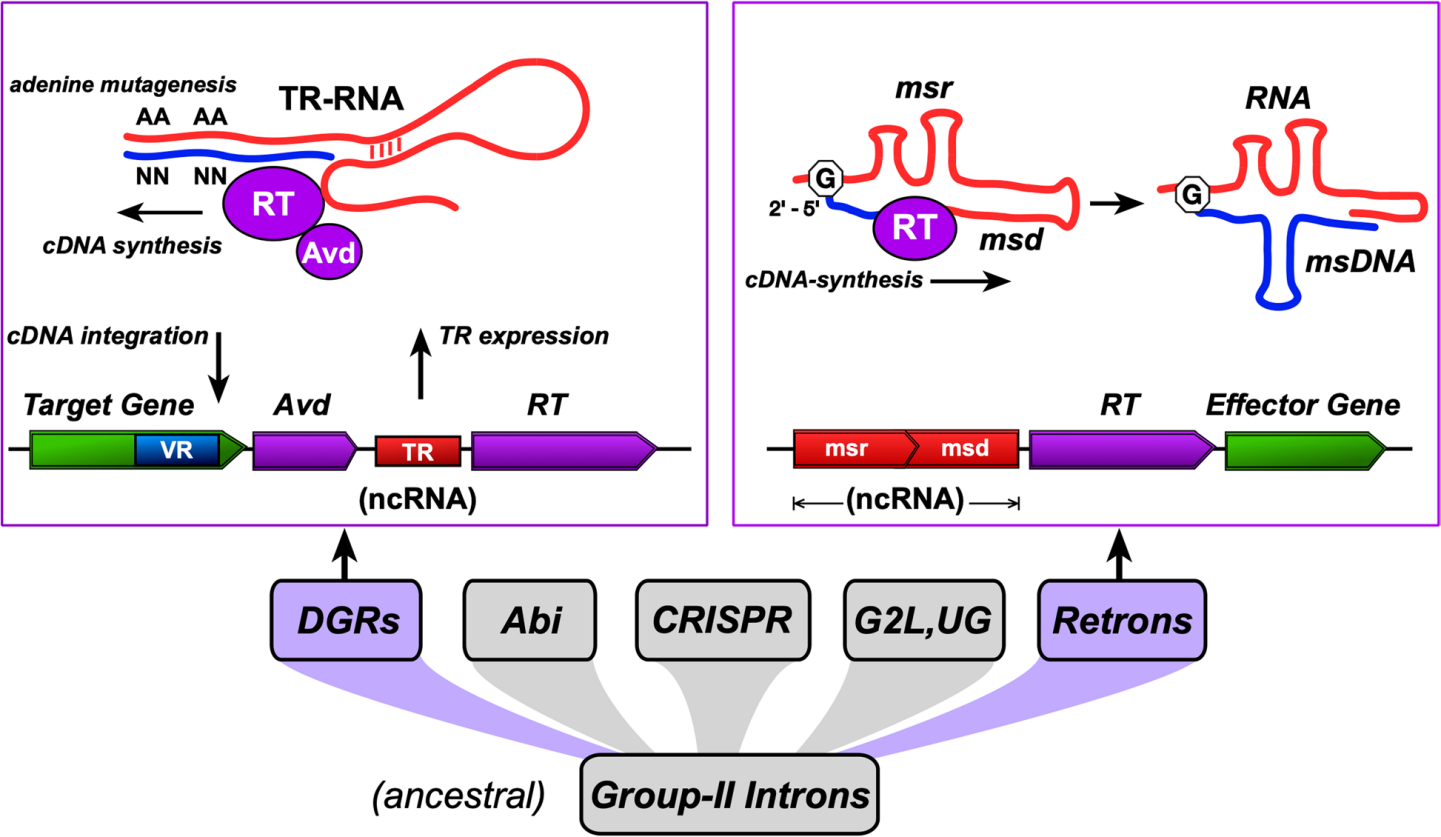
Overview of the molecular mechanisms of DGRs and retrons. DGRs and retrons are highlighted among other modern retroelements, with group-II introns shown as the ancestral progenitor. A prototypical DGR is depicted with the stages of adenine mutagenesis and retrohoming that involves template (TR) ncRNA (non-coding RNA) expression, cDNA synthesis by the RT-Avd complex, and site-specific integration into the variable repeat (VR) of a target gene. A prototypical retron is shown with the initiation of cDNA synthesis at a branched guanosine in retron msr RNA. The resulting msr-msd molecule may interact with products of one or more effector genes to make up multipartite retron defense systems

The molecular mechanism of DGRs (Fig. 1) involves reverse transcription of a template RNA and selective rewriting of template adenines to random bases, which in turn drives amino acid substitutions in a target gene following integration [13, 14]. In addition to the essential reverse transcriptase (RT) and the invariant template RNA sequence, DGRs may require an accessory gene that coordinates cDNA synthesis and integration (i.e., retrohoming). Moreover, several *cis*-acting sequence features enhance or guide homing, including the initiation of mutagenic homing (IMH) site immediately downstream of the target region(s), and one or more stem-loop features that flank IMH [14].

In the model DGR system of *Bordetella* phage, the template repeat (TR) RNA molecule encodes a short sequence (~ 150 bp) that corresponds to the variable repeat (VR) of a target gene, while the trailing RNA sequence (~ 200 bp) in is predicted to have conserved structure that interacts with reverse transcriptase (RT), in complex with the accessory protein [15, 16]. Recent experimental work with an in vitro system comprising *Bordetella* DGR-RT and Avd provides new insights on the role of adenine mutagenesis during cDNA synthesis by DGRs [17]. Handa et al. demonstrated that misincorporation at RNA template adenines was found to be associated with the low catalytic efficiency of DGR-RT, while also uncovering evidence that mutagenesis depends on template purine bases having either amine or carbonyl groups at the C6 position of adenine vs guanine, respectively. Although a prototype is emerging for the mechanism of mutagenesis in DGR systems, the molecular determinants of target integration remain elusive.

## DGR Distribution and Evolutionary History

Early systematic surveys of metagenomic datasets and available bacterial genomes led to the prediction that DGRs are widespread among bacteria and their phage [1, 18, 19]. More recent environmental studies discovered DGRs in Archaea [20], and as a prominent feature of uncultivated members of candidate phyla from subsurface aquatic ecosystems [21]. These uncultivated bacterial and archaeal lineages are predicted to mainly comprise nano-sized organisms that depend on microbial hosts for molecular resources, to accommodate their biosynthetic deficiencies [22]. Although DGRs are predicted to have a role in diversifying attachment proteins for these putative epibionts, most of their diverse target genes remain entirely uncharacterized. Most recently, all publicly available genomic and metagenomic databases have been examined, resulting in a more complete understanding of DGR distribution across taxonomic, functional, and biogeographic scales [23, 24]. DGRs are especially prevalent in microbial constituents of the human microbiome, where they are particularly enriched in gut microorganisms [23–26]. Through bioinformatic screening, DGRs have been found in numerous viral and cellular genomes derived from human gut samples. DGRs were also identified in gut phage that infect *Bacteroides dorei* [27], and in CrAss-like phage where the target gene—a tail collar fiber protein—is also conserved in phage genomes that lack DGRs [28]. Whereas some DGRs appear to reside in fixed chromosomal loci, they can also occur in phage, prophage, plasmids, and conjugative elements, offering evidence that these elements have dynamic potential for mobility between microbial genomes [23]. This capacity for exchange across taxonomic boundaries complicates an attempt to determine the evolutionary history for these elements.

A comprehensive analysis of cyanobacterial genomes found that DGRs are common to dozens of members in the phylum, while phylogenetic reconstruction for DGR-RT seems to depict horizontal exchange among distantly related taxa [29]. Members of *Trichodesmium*, *Nostoc*, *Anabaena*, and *Calothrix*, among other genera, possess multiple distinct DGR-RT genes that appear to have been obtained independently, perhaps via HGT within the phylum. The closest RT relatives from non-cyanobacterial genomes belong to members of Chlorobi and Chloroflexi [29], which may have been early sources of DGR into cyanobacteria. The absence of DGR-RT from particular clades, such as *Prochlorococcus, Gloeobacter*, or representatives of plastids, further suggests that DGRs were either recently acquired in a subset of cyanobacteria, or their distribution in the phylum is a result of loss in particular lineages.

## DGR functional diversity

**Fig. 2:**
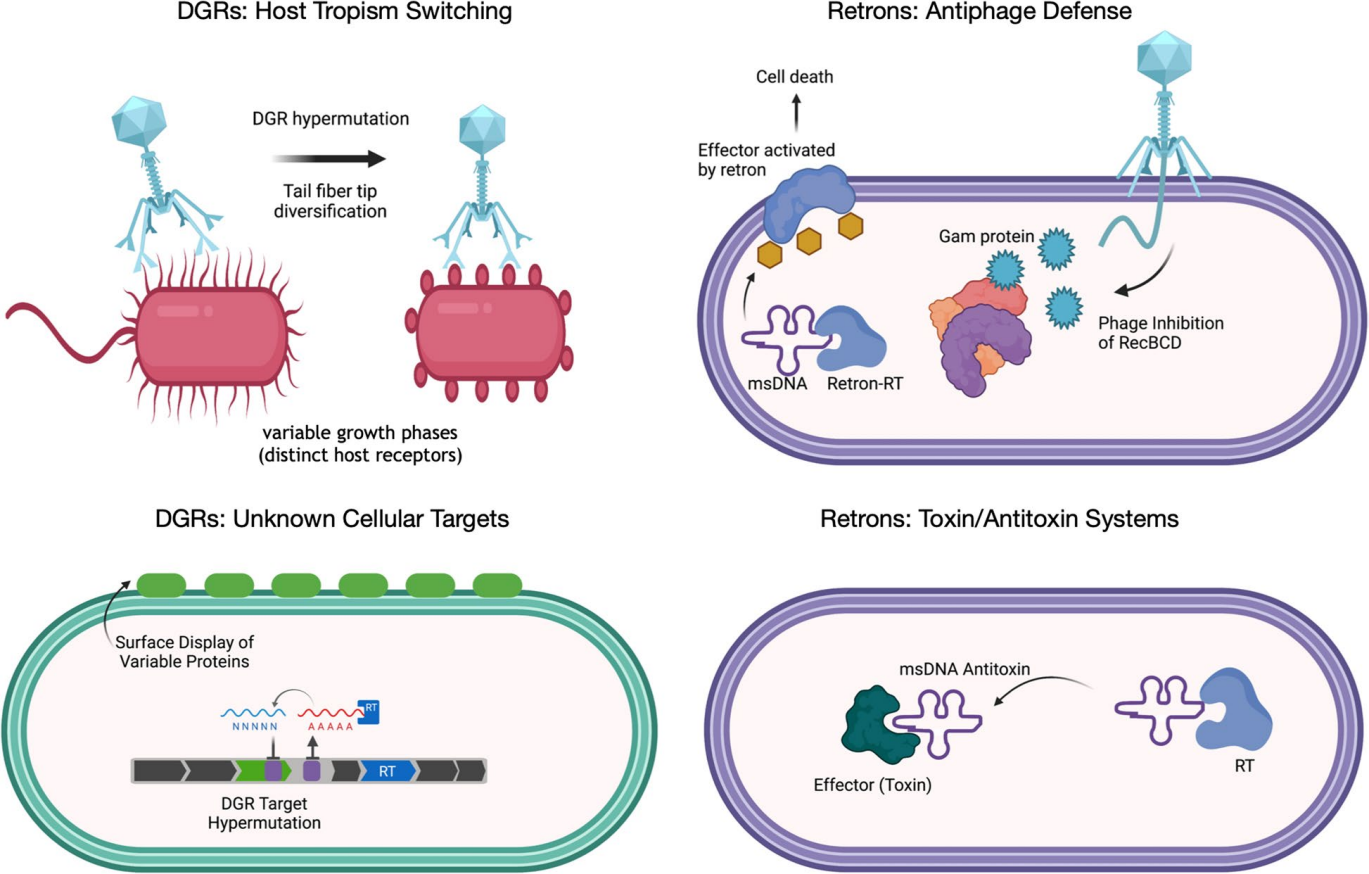
Summary of the functional roles of domesticated retroelements in prokaryotes. The functional significance of DGR hypermutation is depicted in phage that diversify tail tip proteins in order to attach to variable host receptors. Additional cellular DGR targets in *Legionella pneumophila* and *Treponema denticola* encode outer membrane proteins whose specific functions remain unknown. Retrons in *E. coli* have been shown to activate effector proteins that trigger abortive infection and cell arrest. In the case of other bacterial retrons, msDNA may directly inhibit effector proteins, serving as a toxin/antitoxin system. Figure created with BioRender.com

While DGRs were discovered in phage, where they diversify genes for host attachment [1], these retroelements seem to offer broad utility as a modular tool for hypermutation of diverse cellular and viral targets (Fig. 2). Few molecular targets have been experimentally characterized to date, yet prediction from the wealth of microbial genome sequence data suggests that roughly half of DGR-variable proteins appear to have cellular (i.e. non-viral) functions [23, 24]. Hypervariable proteins were characterized in several clinical strains of *Legionella pneumophila*, which appear to actively diversify genes for a lipoprotein that is displayed on the outer membrane of the cell [30]. In the DGR target protein of *Treponema denticola* (TvpA)*,* a lipoprotein signal sequence is predicted to be involved in export to the outer membrane, possibly for attachment to other bacterial or epithelial cells [3]. In the gut microbiome, a diverse array of DGRs appear to have a role in targeting both phage and cellular genes [24–26], yet these variable proteins remain functionally uncharacterized.

With little available experimental data for the vast array of known bacterial and archaeal DGRs, we are limited in understanding the functional significance for most targets of hypermutation. Many DGRs belonging to uncultivated parasites or epibionts that form lineages of candidate phyla are predicted to be involved in modulating host interaction [21]. For cyanobacterial DGR targets, protein domain prediction across DGR targets has shown that fused N-terminal components of the variable CLec-like domain may have an unclear regulatory function [29]. It is speculated that a flexible ligand-binding module of stress response proteins may allow these organisms to rapidly adapt in a changing environment [29]. Another potential advantage of hypervariation might be to mitigate or bypass the function of effector proteins of abortive pathways, such that DGR variation may enable individuals to evade programmed cell death.

## Retrons

Virtually all organisms spanning the tree of life encode a variety of non-coding RNAs in their genomes, including a majority of elements whose functions are still uncharacterized. Retrons were the first retroelements to be discovered from bacteria [31, 32] (and later, archaea [33]) and for years, their functional role in bacterial cells remained elusive [34, 35]. Initially characterized within several model bacterial species, these small DNA molecules are generated via reverse transcription of an ncRNA template, which matures into an RNA–DNA hybrid, often producing many copies in the bacterial cell (Fig. 1). Although the function of retrons and their multi-copy single-stranded DNA (msDNA) was unknown for years since their discovery, recent evidence points to a specific role in abortive defense systems to mitigate phage infection (Fig. 2) [4, 5]. Moreover, new preliminary findings have uncovered a similar biological role for previously unknown groups (UG) and Abi-like retroelements in providing defense against phage infection [36].

From a genomic view, retrons are composed of a reverse transcriptase (RT) gene alongside retron RNA and DNA regions, or *msr* and *msd*, respectively (Fig. 1). A single transcript is produced from the msr-msd-RT sequence and forms an RNA secondary structure that serves as the template for cDNA synthesis following 2’-OH priming at a guanosine residue. The msd RNA sequence is reverse transcribed to msDNA, which is bound to the template msr-RNA at both 5’ and 3’ ends (Fig. 1). Retrons have been experimentally characterized within members of *Deltaproteobacteria*, *Gammaproteobacteria*, *Alphaproteobacteria*, and *Bacteroides* [37], where RT-mediated msDNA synthesis was shown to be a universal property of these elements.

The RNA molecules of retrons are an essential precursor to the formation of RNA–DNA hybrid molecules, which, until recently, had unclear cellular functions. The bacterial retron is typically encoded in a defense island alongside effector genes, including DNA-binding proteins (HTH, zinc-finger, etc.), cold-shock proteins, endonucleases, and proteases [38]. These effectors may be “guarded” or activated by the retron RNA–DNA hybrid molecule, as demonstrated for the *E. coli* retron, Ec48, which confers defense against a broad range of phage [4, 5, 39]. Retrons and their effector proteins are hypothesized to serve as a toxin-antitoxin system (Fig. 2), whereby phage infection leads to abortive cell death, ensuring individual cell sacrifice to the population’s benefit [5]. The anti-phage activity of retrons appears to be dependent on both msDNA structure and functional domains, such as topoisomerase primase (TOPRIM), toll-interleukin-like receptor (TIR), and ATPase and HNH nuclease domains [38, 40]. Given that a diverse array of distinct gene operons are found in several bacterial lineages, while other retrons seem to lack clear association with proximal effector genes [38], it is possible that retrons have been recruited for distinct cellular functions in addition to abortive anti-phage response.

## Retron distribution and evolutionary history

Genomic surveys have estimated the distribution of retrons across bacterial phyla and the most comprehensive efforts explored a database of 9141 non-redundant representatives from approximately 200,000 reverse transcriptase sequences that comprise each retroelement class, along with unknown RT lineages [41, 42]. Retrons identified in microbial genomes are grouped based on their RT phylogeny: 11 clades have been described that are associated with various ncRNA structures and a diverse array of effector genes, or RT-fused domains [38].

Bacterial retrons show evidence of both vertical and horizontal exchange in different genomes. For example, closely related retrons in different strains of myxobacteria have a similar codon usage signature to other conserved myxobacterial genes [43]. By contrast, widespread *E. coli* retrons do not closely match the codon usage of core genes, suggesting that they were recently acquired and horizontally exchanged in the evolutionary history of these enterobacteria [43]. Intriguingly, retrons are commonly found in prophage sequences of bacterial genomes, where phage transduction is a likely driver of their mobility into new recipients [34, 44]. Whereas the experimentally validated prophage retrons are from *E. coli* genomes, prophage-encoded retrons are likely to be found in other bacterial genomes.

An intricate evolutionary history of retroelements has been previously described with group-II introns as the early ancestors from which the other classes emerged [38, 45, 46]. Retrons and DGRs may share a recent common ancestor, given that they have several mechanistic traits in common and are more closely related in comparison with most group-II introns. Key characteristics are shared by retrons and DGRs: i) a single, small RNA transcript is generated, from which a DNA-RNA heteroduplex forms and ii) cDNA synthesis by RT is template-primed. However, retrons seem to lack the ability for integrative retrohoming that DGRs and group-II introns both exhibit, albeit through separate mechanisms [13, 47].

## Genomics and ecology of domesticated retroelements

The last decade has witnessed a tremendous increase in the number of genomes that offer access to the genetic makeup of microbial life. In addition to advances in cultivation strategies that improve conventional means of isolating microbes [48–50], single-amplified genomes and metagenome-assembled genomes have provided additional means to access genomes from branches of life that have been difficult to cultivate in laboratory environments [51–54]. Increasingly available long-read sequencing technologies [55] predict that the number, breadth, and quality of microbial genomes will continue to improve.

To date, comparative genomic investigations into DGRs and retrons have emphasized identification and classification using homology-based tools for feature detection [19, 21, 23, 24, 38, 46, 56]. These approaches have recently enabled systematic identification of thousands of new retroelements that define a taxonomic and biogeographic distribution, as well as functional diversity of associated protein families [24, 38]. The utility of genomes, however, does not extend into capturing the ecological and evolutionary significance of retroelements and their dynamic nature in naturally occurring microbial populations. A genome typically represents an individual member of a population and does not offer immediate access to dynamic regions wherein the activity, diversity, or mobility of specialized mechanisms may not be uniform across all members of an environmental population. Yet, the shortcomings of individual genomes to understand genomic dynamism of closely-related environmental populations can be at least partly solved by metagenomic read recruitment. Indeed, metagenomic read recruitment strategies are used not only to understand biogeography of individual taxa across environments [57–59], but also to elucidate genetic variation within individual environmental populations [60, 61], enabling deeper insights into population dynamics across temporal and spatial scales as a function of environmental change.

The increasing availability of genomes, metagenomes, and computational strategies presents a new frontier to approach the dynamic yet understudied landscape of genetic variants that emerge within naturally occurring microbial populations due to the activity of domesticated retroelements [21, 24, 62]. Here we offer a glimpse into those opportunities by surveying marine populations of *Trichodesmium erythraeum* using an isolate genome and marine metagenomes.

**Fig. 3:**
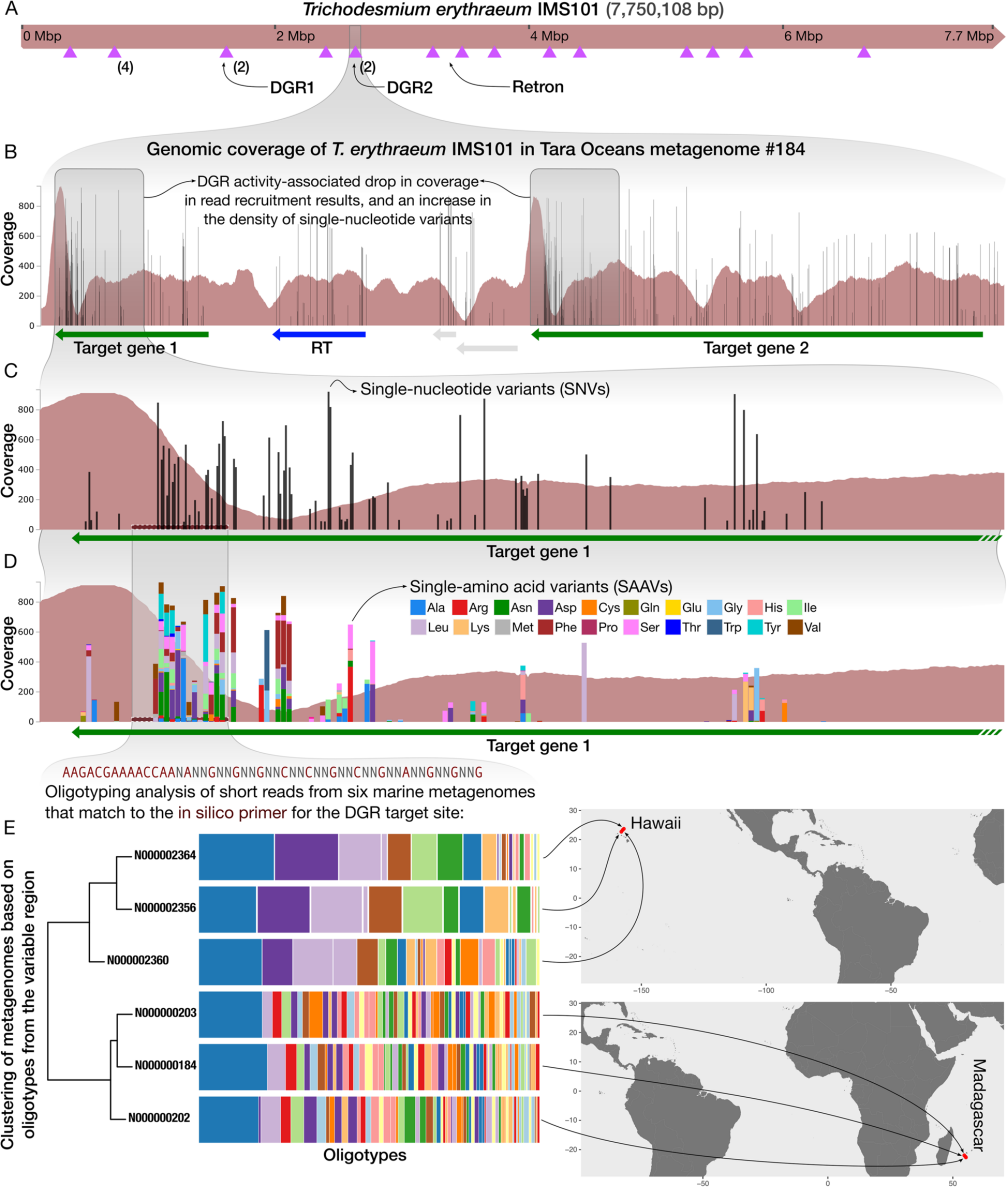
A bioinformatics workflow to survey DGR activity and ecology in metagenomes. **A** Genome map of *Trichodesmium erythraeum* strain IMS101, indicating the positions of two DGRs, one retron, and several DGR target genes (purple triangles). **B** An anvi’o [68] visualization of the coverage of a section of the *T. erythraeum* genome with short reads recruited from the Tara Oceans Project metagenome N000000184, and the distribution of single-nucleotide variants (SNVs). **C** A detailed representation of the decrease in coverage and increase in the SNV density in Target Gene 1, which indicates the presence of DGR activity in environmental populations. **D** Distribution of single-amino acid variants (SAAVs) in the same region of the gene. SAAVs are calculated based on the frequency of codons found in metagenomic short reads that fully map to a given codon position in the gene [61], thus, they are much more effective to quantify the extent of non-synonymous variation environmental populations of *Trichodesmium* have accumulated. **E** Oligotyping analysis of all metagenomic short reads that fully match to the primer sequence, location of which is indicated by the horizontal bar in panels **C** and **D**. The sequence pattern takes into consideration conserved and variable nucleotide positions as revealed by SNVs in read recruitment results. Metagenomic short reads that match the pattern from each sample were subjected to an oligotyping analysis [69], during which high-entropy nucleotide positions divide sequences into distinct, ecologically relevant groups. While the bar plots visualize the oligotype profiles per sample and their diversity, the dendrogram on the left shows how samples relate to each other based on Jaccard Similarity. Finally, arrows point to the sampling sites near Hawaii and Madagascar that correspond to metagenomes that were used in this analysis on a world map

Surveys of retroelements in reference genomes suggest that the phylum Cyanobacteria is the most enriched with putative DGRs and retrons [23]. *Trichodesmium*, a genus of marine cyanobacteria, includes lineages that are widespread and abundant in oligotrophic tropical and subtropical oceans [63], and that contribute to the biogeochemical cycling of Nitrogen in marine habitats [64, 65], such as *T. erythraeum*. Pfreundt et al. demonstrated that template RNAs of DGRs are highly expressed in *T. erythraeum* IMS101 [66], however, targeted mutagenesis was not detectable in laboratory cultures [66]. To investigate the ecology of retroelements of environmental *T. erythraeum* populations, we used the isolate genome *Trichodesmium erythraeum* IMS101, which contains at least 10 distinct reverse transcriptase genes, including two DGR-RTs and one retron (Table S1, Fig. 3), and six metagenomes from the Tara Oceans Project [67], in which *T. erythraeum* was abundant (Table S2).

The genome consists of several reverse transcriptase genes in dispersed loci (Table S1), including two DGR-RTs (Tery_1035 and Tery_1728), and one retron RT (Tery_2145), as previously noted [29, 38, 66]. Each of the DGRs have two proximal DGR target genes (Fig. 3A), in addition to remote target genes that were previously described [29, 66]. Of the 18 loci in which we found DGR-TR/VR homology, at least 11 showed a pattern of localized hypervariability as revealed by metagenomic read recruitment results (Table S3). In metagenomic read recruitment results, localized hypervariability is often manifested by a sharp drop in coverage and an increase in single-nucleotide variants (SNVs) due to the decreasing identity of environmental sequences to the reference genome as a likely outcome of DGR activity. Fig. 3B demonstrates this phenomenon in the context of DGR2 target genes. Variation in coverage and SNVs are effective indicators of within-population hypervariability in read recruitment results (Fig. 3C), however, SNVs are not sufficient indicators of change that influence amino acid composition [61]. Yet this information can be recovered through the analysis of single-amino acid variants (SAAVs), which represent the allele frequency of amino acids in a single codon position based on the number of short reads that fully cover the codon. Even though the vast majority of non-synonymous diversity within a codon position is typically represented by two amino acids [61], the SAAVs that correspond to the same DGR2 target region in *T. erythraeum* revealed a substantial amount of diversity in amino acids (Fig. 3D), which can confirm the known mechanism of DGR-mediated sequence evolution in metagenomic read recruitment results. Another important question is whether the observed variation is associated with the biogeography of a given population. This question, too, can be approached via metagenomic read recruitment results. In depth characterization of hypervariable regions requires one to work with raw short reads, since read recruitment results will exclude many short reads due to their low identity even if they are coming from a homologous region of the genome. An option is to identify a pattern of conserved and variable nucleotides by analyzing SNVs found read recruitment results, and search for reads that match to this ‘in silico primer’ in quality-filtered paired-end short reads. Identifying such reads for *T. erythraeum* and performing an oligotyping analysis on them showed a clear distinction between metagenomes collected from Hawaii and Madagascar based on the putative DGR activity (Fig. 3E). This analysis demonstrates a bioinformatics workflow that can uncover dynamic ecological and evolutionary patterns of retroelements by employing metagenomics (the URL https://merenlab.org/dgrs-in-metagenomes details the steps of data generation and visualization for similar applications). Combined with short-interval longitudinal sampling, this approach could help identify and characterize patterns of rapid evolution within naturally occurring microbial populations in any habitat.

## Conclusions

Both DGRs and retrons are enigmatic genetic elements, as much remains undiscovered in terms of their specific cellular functions, molecular mechanisms, and activity in natural ecosystems. Moreover, the evolutionary history of these beneficial retroelements is far from resolved, though understanding this complex problem will lead to exciting revelations about microbial adaptation to biological conflict and environmental stress. Functional genomic experiments may shed light on new biological roles for DGRs or retrons, beyond the existing paradigms on anticipatory variation in host attachment, or dynamic antiphage defense. New computational tools are being applied to multi-omics datasets in order to study the ecological and evolutionary dynamics of DGRs and retrons across different biomes. To this end, a focused temporal examination of individual genomes, or pangenomes, may lead to key insights about the significance of retroelements to specific microorganisms.

## Supplementary Information

**Additional file 1:** **Table S1.** Reverse transcriptase genes in Trichodesmium erythraeum IMS101 and the coordinates of their associated retroelement features (where applicable). **Table S2.** Coordinates of putative remote DGR target genes, with the corresponding template repeat (TR) based on homology. **Table S3.** Metagenome details for TARA Global Oceans datasets that were used in the Trichodesmium pangenomic analysis. 

## Data Availability

Data sharing not applicable to this article as no new datasets were generated or analysed during the current study.
